# Increased Noise in Cortico-Cortical Integration After Mild TBI Measured With the Equivalent Noise Technique

**DOI:** 10.3389/fneur.2019.00767

**Published:** 2019-07-18

**Authors:** Tatiana Ruiz, Alex S. Baldwin, Daniel P. Spiegel, Robert Hess, Reza Farivar

**Affiliations:** ^1^Research Institute of the McGill University Health Center, Montreal, QC, Canada; ^2^Vision Sciences, Essilor R&D, Center for Innovation and Technology, Singapore, Singapore

**Keywords:** traumatic brain injury, internal noise, efficiency, contour perception, equivalent noise method, cortical integration

## Abstract

The bulk of deficits accompanying mild traumatic brain injury (mTBI) is understood in terms of cortical integration—mnemonic, attentional, and cognitive disturbances are believed to involve integrative action across brain regions. Independent of integrative disturbances, mTBI may increase cortical noise, and this has not been previously considered. High-level integrative deficits are exceedingly difficult to measure and model, motivating us to utilize a tightly-controlled task within an established quantitative model to separately estimate internal noise and integration efficiency. First, we utilized a contour integration task modeled as a cortical-integration process involving multiple adjacent cortical columns in early visual areas. Second, we estimated internal noise and integration efficiency using the linear amplifier model (LAM). Fifty-seven mTBI patients and 24 normal controls performed a 4AFC task where they had to identify a valid contour amongst three invalid contours. Thresholds for contour amplitude were measured adaptively across three levels of added external orientation noise. Using the LAM, we found that mTBI increased internal noise without affecting integration efficiency. mTBI also caused hemifield bias differences, and efficiency was related to a change of visual habits. Using a controlled task reflecting cortical integration within the equivalent noise framework empowered us to detect increased computational noise that may be at the heart of mTBI deficits. Our approach is highly sensitive and translatable to rehabilitative efforts for the mTBI population, while also implicating a novel hypothesis of mTBI effects on basic visual processing—namely that cortical integration is maintained at the cost of increased internal noise.

## Significance Statement

Traumatic brain injury symptoms are largely understood in terms of neuronal and axonal loss, reflected in deficits that are largely understood in terms of cortical integration. An untested idea is that integration is maintained and compensated, but that injury causes increased computational noise. We tested this hypothesis using a psychophysical task with a strong neurophysiological basis that requires cortical integration and utilized an established approach to separately estimate internal noise and integration efficiency. Our results demonstrate that injury increases noise in cortical circuits without affecting integration efficiency. This sensitive and informed approach has important implications for diagnosis and rehabilitation of the two million U.S. patients affected annually by traumatic brain injury.

## Introduction

Traumatic brain injury (TBI) affects over 2,000,000 people in North America every year, with a sizeable portion of patients continuing to report deficits of attentional, mnemonic, or sensory nature many months after injury (1–5). The cognitive deficits of mild TBI (mTBI) can be present across different types or modes of injury, suggesting them to be general in nature (6). These deficits are often interpreted as a decreased capability of the cortical system to integrate information after injury.

Loss of tissue could have two distinct effects on the performance of a system—it could impair the integrative capacity of the system by reducing the efficiency with which information is processed, or it could increase the internal noise of the system. Here we aim to assess whether cortical changes caused by mTBI increase noise or decrease integration, or both.

Of the domains potentially affected by mTBI, the cortical visual system is the most characterized and best understood—the human visual system has high homology to multiple animal models, and over 50 years of neurophysiology and psychophysics make it the most characterized cortical system (7, 8). Visual complaints are common after mTBI (9), and we and others have successfully used visual psychophysics to quantify cortical visual deficits caused by mTBI (10–14). The availability of highly sensitive psychophysical methods with physiologically-motivated computational models behind them make vision an excellent platform for characterizing and understanding cortical changes that follow mTBI.

High-level deficits such as memory and attentional losses can be broadly described as impairments of cortical integration over large cortical scales (15–17). A highly controllable model of cortical integration is contour integration—the perception of a shape through pooling of local edge segments that together describe a shape (18, 19). Contour integration is a crucial step in the processing of visual shape representation and is understood to require well-characterized integrative mechanisms at the lowest levels of the cortical visual hierarchy (20). Recently, a new contour integration approach has been developed that has the capability to allow measurements of both efficiency and internal noise (21) something not attainable from the original approach of Field et al. (18).

We therefore measured cortical integrative capacity and noise using the tightly-controlled visual contour integration task (21). Importantly, we can quantify both the capability of the cortical integration process that occurs for contours, as well as the amount of “noise” that is limiting the system's performance. To enhance our sensitivity to changes in integrative capacity and/or internal noise, we made our measurements independently for the four visual quadrants, which simultaneously enabled us to probe previously-reported visual field biases in mTBI (12, 22).

## Materials and Methods

### Participants

All participants gave their informed consent prior to taking part in the experiment. All procedures were in accordance with the Code of Ethics of the World Medical Association (Declaration of Helsinki) and were approved by the Research Ethics Board of the McGill University Health Center.

All participants were screened for anomalous vision loss or vision disorders (glaucoma, retinal detachment, macular degeneration, etc.). They had normal or corrected to normal visual acuity (wore their usual refractive correction). The average age of the participants was 39.7 years old (SD = 14.4 years, *n* = 56) in the mTBI group and 35.5 years old (SD = 13.8 years, *n* = 24) in the control group.

#### TBI Group

Participants (Table 1) were recruited through the McGill University Health Center out-patient TBI clinic. The diagnostic criteria for mild TBI were: Glasgow Coma Scale score between 13 and 15, <30 min of loss of consciousness, and <24 h of amnesia regarding events immediately before or after the accident. Patients with mild TBI who gave their authorization to be contacted went through a phone screening interview. The exclusion criteria were (1) family history of epilepsy or seizure, or the administration of prescription medication with increased risk of seizure, (2) severe tremors or involuntary movements, (3) general anesthesia in the past 6 months, (4) mTBI occurred <1 month ago or more than 2 years ago, (5) presence of a brain lesion, (6) a history of multiple brain injury. During validation of patient's clinical history, we found that five of them had had previous head traumas, with their last one being a mild TBI (GSC 13–15). We removed these five subjects from our analysis, but their data were not discarded and instead, we analyzed them separately. We did not exclude participants on the basis of having received an intervention or not. Following our previous publication, participants filled a questionnaire adapted from Assessment with Mild Traumatic Brain Injury for the Defense Centers of Excellence for Psychological Health and Traumatic Brain Injury (13) investigating blurred vision, migraines, behavioral change to palliate visual discomfort etc. The final sample size of tested mTBI participants was 55 (13 males and 42 females), with an additional five polytrauma participants (two males, three females).

**Table 1:** Participants.

**Subject**	**Age**	**Gender**	**TMT time**	**TMT errors**	**Bells time**	**Bells missed**	**Education Level**	**Handedness**	**Diagmsis**	**Loss of consciousness**
t1	59	M	29.699	0	66.38	5	11th Grade	Right	Mild complex	Yes
t2	56	F	35.07	0	126.163	0	11th Grade	Right	Mild simple	No
t3	57	M	111.16	0	116.11	6	Bachelor's Degree	Right	Mild	Yes
t4	33	M	23.107	0	89.576	10	Bachelor's Degree	Right	Mild simple	Yes
t5	57	F	20.779	0	150.143	2	Master's Degree	Right	Mild	Yes
t6	58	M	29.117	1	67399	0	Master's Degree	Right	Mild simple	No
t7	54	F	25.989	0	115.183	1	Bachelor's Degree	Right	Mild	Yes
t8	40	M	16.01	0	65.93	0	Bachelor's Degree	Right	Mild	No
t9	64	M	24.6	0	80.29	5	11th Grade	Left	Mild	No
t10	38	F	38.2	0	115.1	2	11th Grade	Right	Mild	No
t11	38	F	33.646	0	82.928	6	11th Grade	Right	Mild simple	Yes
t12	31	F	20.842	1	61.49	9	Doctoral Degree	Right	Mild complex	Yes
t13	23	F	26.58	0	131.79	0	Bachelor's Degree	Right	Mild simple	No
t14	32	F	21.84	0	94.18	1	11th Grade	Right	Mild simple	Yes
t15	55	F	39.204	0	80.945	7	Bachelor's Degree	Right	Mild simple	Yes
t16	55	F	37.65	0	117.786	2	Bachelor's Degree	Right	Mild simple	No
t17	53	F	22.019	0	77.569	4	Bachelor's Degree	Right	Mild trivial	No
t18	32	F	26.398	0	107.426	5	Doctoral Degree	Right	Mild simple	No
t19	41	F	15.442	0	74.9	1	Bachelor's Degree	Right	Mild	Yes
t20	18	F	25.933	0	76.599	3	11th Grade	Right	Mild simple	Yes
t21	50	F	23.369	0	68.446	6	Professional DEC	Right	Mild simple	Yes
t22	20	F	27.62	0	50.909	13	General DEC	Right	Mild simple	Yes
t23	22	F	14.8	0	60.5	14	Bachelor's Degree	Left	Mild simple	No
t24	46	F	48.862	0	103.052	2	Bachelor's Degree	Right	Mild simple	No
t25	19	F	19.98	0	46.6	6	11th Grade	Right	Mild complex	Yes
t26	41	F	36.64	1	127.91	5	Professional DEC	Left	Mild	No
t27	69	F	31.57	0	122.43	1	Professional DEC	Left	Mild simple	No
t28	61	M	58.65	0	101.05	3	Bachelor's Degree	Left	Mild	Yes
t29	34	F	32.72	0	93.83	4	11th Grade	Right	Mild simple	Yes
t30	56	F	26.94	0	70.08	6	Master's Degree	Right	Mild simple	No
t31	29	F	27.62	0	38.09	10	Bachelor's Degree	Right	Mild simple	Yes
t32	33	F	20.23	0	71.09	5	Master's Degree	Right	Mild simple	No
t33	57	F	27.33	0	75.3	7	Bachelor's Degree	Left	Mild simple	Yes
t34	32	F	25.72	0	88.5	1	Profession al DEC	Right	Mild simple	Yes
t35	63	F	22.14	0	89.19	1	11th Grade	Right	Mild complex	No
t36	18	F	23.11	1	101.84	1	11th Grade	Right	Mild simple	No
t37	40	M	26.53	1	88.21	1	Master's Degree	Right	Mild simple	Yes
t38	23	F	22.2	0	32.18	5	Bachelor's Degree	Right	Mild simple	Yes
t39	44	F	19.93	1	62.08	11	Bachelor's Degree	Right	Mild simple	No
t40	24	F	33	1	78.48	0	Bachelor's Degree	Left	Mild simple	Yes
t41	31	F	23.28	0	119.65	2	Bachelor's Degree	Right	Mild	Yes
t42	28	M	32.35	1	65.43	7	General DEC	Right	Mild complex	Yes
t43	24	M	28.28	0	87.75	3	Bachelor's Degree	Right	Mild simple	Yes
t44	28	F	20.55	0	46.37	7	General DEC	Right	Self-reported	Yes
t45	44	M	22.35	0	88.36	1	Bachelor's Degree	Right	Mild simple	No
t46	19	F	13.28	0	78.84	2	General DEC	Right	Self-reported	No
t47	37	F	22.53	0	62.68	2	11th Grade	Right	Mild	Yes
t48	27	F	31.26	0	122.4	1	Bachelor's Degree	Right	Mild simple	Yes
t49	24	M	35.49	1	183.07	0	General DEC	Right	Mild simple	Yes
t50	45	F	30.21	0	153.87	0	General DEC	Right	Mild simple	No
t51	53	M	35.63	1	138.45	0	Master's Degree	Right	Mild simple	Yes
t52	39	F	24.58	0	61.93	2	Bachelor's Degree	Right	Mild simple	No
t53	50	F	22.25	0	67.93	3	Bachelor's Degree	Right	Mild simple	No
t54	20	M	16.8	1	77.47	3	General DEC	Right	Mild simple	Yes
t55	40	F	43.1	0	171.8	1	Bachelor's Degree	Right	Self-reported	n/a
poly1	24	M	24.5	0	122.57	1	Bachelor's Degree	Right	Multiple	n/a
poly2	18	F	14.3	0	40.74	4	Master's Degree	Right	Multiple	n/a
poly3	26	F	37.92	0	124.68	2	n/a	Right	Multiple	n/a
poly4	49	F	36.34	0	98.31	10	Bachelor's Degree	Right	Multiple	n/a
poly5	23	F	33.44	0	110.39	1	Bachelor's Degree	Right	Multiple	n/a
c1	42	F	n/a	n/a	68.345	1	Doctoral Degree	Right	None	n/a
c2	40	F	n/a	n/a	76.398	4	GEP General DE	Right	None	n/a
c3	53	F	n/a	n/a	73.679	7	Master's Degree	Left	None	n/a
c4	70	M	n/a	n/a	108.24	2	Bachelor's Degree	Right	None	n/a
c5	19	M	n/a	n/a	68.66	1	n/a	n/a	None	n/a
c6	54	M	n/a	n/a	92.8	1	Master's Degree	Right	None	n/a
c7	49	F	n/a	n/a	80.239	1	EP Professional	Right	None	n/a
c8	18	F	34.87	0	65.58	7	11th Grade	Right	None	n/a
c9	25	M	18.25	0	48.4	7	Bachelor's Degree	Right	None	n/a
c10	21	F	34.91	0	81.69	6	Bachelor's Degree	Right	None	n/a
c11	53	F	36.48	1			Bachelor's Degree	Right	None	n/a
c12	42	M	12.92	0	57.43	1	Bachelor's Degree	Right	None	n/a
c13	27	M	21.12	0	65.05	2	Bachelor's Degree	Right	None	n/a
c14	50	F	21.15	0	42.39	6	n/a	n/a	None	n/a
c15	20	F	28.32	0	56.76	1	GEP General DE	Left	None	n/a
c16	26	M	21.54	0	46.37	5	Mai.er1S Degree	Right	None	n/a
c17	35	F	23.55	0	77.94	1	Doctoral Degree	Right	None	n/a
c18	46	M	12.63	0	66.11	3	Bachelor1s Degree	Right	None	n/a
c19	25	M	16.85	0	72.67	3	11th Grade	Right	None	n/a
c20	22	F	19.1	1	687	0	Bachelor's Degree	Right	None	n/a
c21	28	M	23.53	0	62.98	4	Doctoral Degree	Right	None	n/a
c22	27	M	15.16	1	48.19	3	Master's Degree	Right	None	n/a
c23	33	M	32.72	0	126.14	0	Doctoral Degree	Right	None	n/a

#### Control Group

Healthy participants were recruited through public announcements in the Montreal General Hospital and on social media. Demographics of the mTBI sample population were evaluated and the control group was sampled accordingly. Exclusion criteria included conditions 1–4 outlined above, and no history of any acquired brain injury. The control group was comprised of 23 individuals (12 males and 11 females). Despite the unequal proportions of males and females in both groups, sex had no effect on any of the LAM parameters, neither when taken as an average nor when assessed individually per quadrant (*p* > 0.05) and was therefore ruled out as a potential extraneous variable.

### Supplementary Evaluation

The Trail Making Test B (23), the Bells Test (24), and the clock-drawing test (25) were administered to mTBI participants to assess visual attention and spatial neglect. All participants responded normally on these tests. Monocular and binocular visual acuity was measured with a Snellen chart at four meters (Logarithmic Visual Acuity Chart; Precision Vision, Lasalle, IL, USA) and their ocular dominance was assessed using the Miles test. Maddox rod, cover/uncover and alternating-cover tests were performed to detect presence of strabismus. Participants were excluded from the study if a strabismus was found.

### Display

Stimuli were produced using Psychtoolbox (26) through MATLAB® (2014b, The Math Works Inc., Natick, Massachusetts) (27) and presented on a gamma-calibrated LG Flatron 915FT Plus monitor using a 10-bit graphics card (Nvidia Quadro 2000). Calibration was done using a photometer, and the mean luminance was 62 cd/m^2^. Subjects were placed consistently at a 77 cm viewing distance from the monitor, with a spatial resolution of 96 pixels per degree of visual angle.

### Stimuli and Procedure

**Figure 1:**
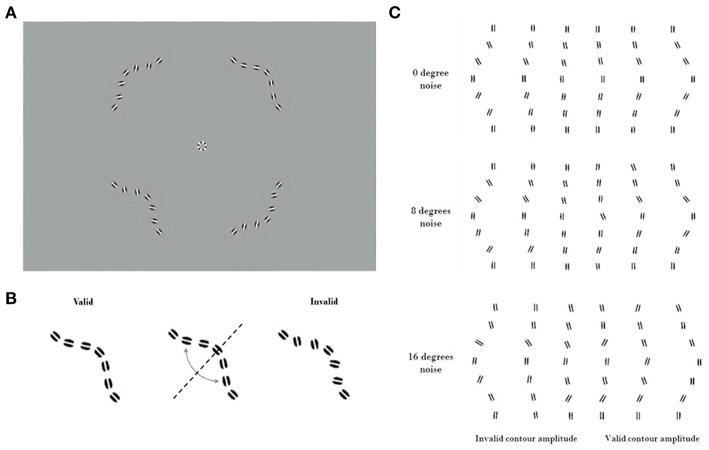
Visual stimuli. Contrast has been enhanced for illustration purposes. **(A)** An example trial of the Good continuation discrimination task, the upper right quadrant contains the valid contour (0° noise). **(B)** Construction of the invalid contour by inverting elements across the valid contour diagonal. Because of the systematic nature of this process, it is not to be confused with the addition of orientation noise. **(C)** Cartoon showing valid and invalid contours varying in amplitude under noise levels 1, 2, and 3 (respectively 0°, 8°, and 16° of SD of orientation noise). Note the increasing difficulty of discriminating between the valid (on the right) and invalid (on the left) contours as amplitude decreases.

Subjects fixated on a marker at the center of the screen. On each trial, four contours appeared simultaneously. Each contour was centered in one quadrant of the visual field, at an eccentricity of 2.8° from fixation. Each contour was comprised of seven log-Gabor wavelets (28) resting on an invisible curved path. The wavelets had a peak spatial frequency of 6 c/deg with a bandwidth of 1.6 octaves. They were presented in cosine phase (white bar with dark flanks) and had an orientation bandwidth of ±25°. The full-width at half-magnitude of the wavelet envelopes measured 1.17 cycles along the stripes, and 0.91 cycles across them. For the target the orientation of each wavelet was aligned with the path of the contour. In the three distractors, the orientations of the wavelets were consistent with a contour curving in the opposite direction. Discriminating the target from the distractors required the subject to combine the orientation and position information of the wavelets (Figure 1).

The contour paths that specified the wavelet locations had the same amplitude for the target and for the three distractors. For large curvature amplitudes the task is easier, as the target appears to be a smoother contour than the distractors. Stimuli were presented for 400 ms, and subjects selected the smoother contour (with “good continuation”) (29) in a four-alternate forced choice (4AFC) task (Figure 1). This task was chosen to ensure data could be collected efficiently from inexperienced subjects (30). The amplitude of the curvature was modulated through a performance-dependent staircase (2-down 1-up), converging at an amplitude where the subject selected the target 70% of the time. The staircase was terminated after 40 trials or following 12 reversals. Thresholds for identifying the smooth contour were obtained using psychometric function fitting (see below). Thresholds were obtained both for stimuli without any added external noise, and for stimuli where the orientations of the individual wavelets were randomly jittered. Measuring performance at different levels of external noise allows the equivalent internal noise and processing efficiency of the contour integration system to be characterized. This method has been previously validated, with human performance measurements quantified compared to that from the ideal observer (21).

We measured discrimination thresholds at three levels of orientation noise: 0°, 8°, and 16°. The orientation of each wavelet was resampled from a Normal distribution centered on its original value (aligned with the contour for the target stimuli, and consistent with a contour of opposite curvature for the distractors). The standard deviation of the Normal distribution controlled the level of external orientation noise. We divided data collection into separate blocks for each noise level (10–15 min each). The order of these blocks was randomized across participants. We have created an interactive illustration of the procedure and corresponding psychometric performance hosted at http://www.farivarlab.com/stimuli-software.

### Experimental Design and Statistical Analysis

#### Experimental Design

We utilized a 2 × 2 × 2 factorial design, with a between-subjects factor (mTBI vs. controls) and two within-subject factors of vertical visual field (upper vs. lower) and horizontal visual fields (left vs. right). To make inferences of differences in internal noise and efficiency across the subjects and quadrants, we analyzed these parameters as estimated by the Linear Amplifier Model (see below) using non-parametric tests (31). To make inferences about quadrant biases, we used the rank assignment of each quadrant for internal noise and efficiency and carried out the same non-parametric tests on these rank values.

#### Statistical Analysis

mTBI subjects tend to be heterogenous and their performance often does not follow a normal distribution—something we have previously observed (13, 14). Our data here also were not normally distributed, and we therefore carried out all our analyses using non-parametric inferential tests, which are more conservative and do not depend on normality of the data distribution and here report the Wald-type statistic (WTS) estimated using the nparLD (31) package in the R Statistical Package (32), which is a non-parametric analog of the repeated-measures factorial ANOVA.

#### Data Pre-processing and Psychometric Fitting

Psychometric function fitting was performed using the Palamedes toolbox (33). The number of trials at each curvature amplitude, and the number of correct responses for each amplitude were fitted with a Gumbel psychometric function. The guessing rate parameter was fixed at 25% (guessing rate for a 4AFC task). The lapse rate was allowed to vary from 0 to 5%, while the threshold and the slope were allowed to vary across noise levels and quadrants.

The equivalent noise method, borrowed from engineering (34–37), uses external noise added to the input of a system to measure the equivalent internal noise level within the system. When the external noise is much smaller than the equivalent internal noise then its effects will be negligible. As the external noise is increased it will reach a point where its effects exceed those of the equivalent internal noise. Beyond this point the external noise will dominate performance, making the system's equivalent internal noise no longer the limiting factor. Psychophysically, noise masking experiments typically find that thresholds are unaffected by low levels of external noise. Beyond some critical value however, the thresholds increase in proportion to the standard deviation of the masking noise. The simplest model for analyzing data from equivalent noise studies is the Linear Amplifier Model (LAM), which has two parameters

(1) $$\begin{array}{l} A_{\text{threshold}}=\frac{\sqrt{\sigma_{\text{external}}^{2}+\sigma_{\text{internal}}^{2}}}{\beta }. \end{array}$$

This predicts a threshold *A*_*threshold*_ for each external noise level σ_*external*_. The fitted σ_*internal*_ parameter indicates the point at which the system transitions from being dominated by internal noise to being dominated by external noise. This is taken as the external noise level that is *equivalent* to the internal noise level. The second fitted parameter β indicates the processing efficiency of the system (38). Elevated internal noise will affect thresholds when the external noise is low or absent but will not change behavior once external noise is greater than internal noise. Reduced efficiency however will increase thresholds at all external noise levels. In the context of our contour task, internal noise indicates the inherent internal limitations affecting the representation of each wavelet, while the efficiency is the capability of the system to combine all of that noisy information to detect good continuity. Thus, the LAM model effectively captures the two key dimensions of performance that we aimed to measure.

**Figure 2:**
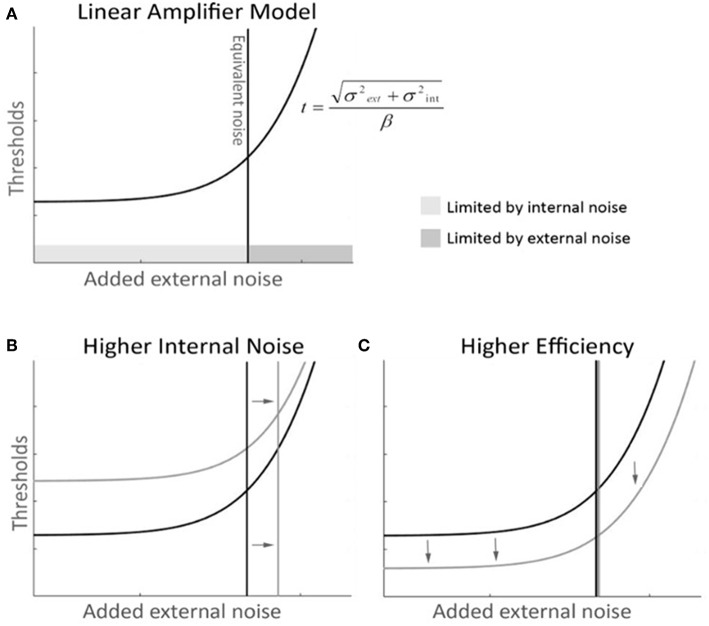
Linear Amplifier model (LAM) graphical description with mock-data. **(A)** The LAM function describes the dynamics of performance thresholds along levels of added external noise—thresholds (t), as a function of external noise (σ_*ext*_), internal noise (σ_*int*_), and efficiency (β). At low levels of external noise, performance is not dependant on external noise and remains constant and limited by internal noise. After the equivalent noise point, additional external noise shifts thresholds upwards, and becomes the major limiting factor of performance. **(B)** A higher internal noise curve (in gray) with unchanged efficiency shows a shift in the equivalent noise point toward higher noise. The thresholds are shifted up, as the tail of the function asymptotes toward the same slope. **(C)** A higher efficiency curve (in gray) with unchanged internal noise shows a global shift toward lower thresholds and maintains the same equivalent noise point.

The LAM was then fitted to the discrimination thresholds to determine internal noise and efficiency (Figure 2). Outlier participants were removed if one of their LAM parameters was further than 1.5 interquartile below Q1 or above Q3 for each group (39, 40), leaving 21 controls (two outliers) and 50 mTBI subjects (five outliers).

Following data collection, we noted that the highest amplitude of curvature produced contours that were difficult to discriminate for several participants in both groups. This was true even at low noise levels. We designed an unbiased means of eliminating these points and validating that our procedure did not bias the results. The data points collected at these amplitudes were unreliable (they resulted in non-monotonic psychometric functions). We removed these data points if doing so significantly improved the fit of the Gumbel function to the data, as determined by a Chi-square goodness-of-fit test. Eleven control subjects and 20 mTBI subjects had points removed. Within-subject comparison of before and after the outlier removal showed no significant difference across groups for all quadrants and all noise levels which means that the group differences found after fitting the LAM were not biased by our outlier removal procedure (*WTS* 0.89 *p* > 0.3). The unbiased preprocessing step significantly decreased variability across the pool of all participants across both groups for quadrants 1, 2 and 3 (*WTS* 8.5 *p* = 0.0035, *WTS* 4.4 *p* = 0.035, *WTS* 9.57 *p* = 0.002) and for noise level 2 (*WTS* 6.67 *p* = 0.01).

## Results

### Higher Internal Noise Following TBI

**Figure 3:**
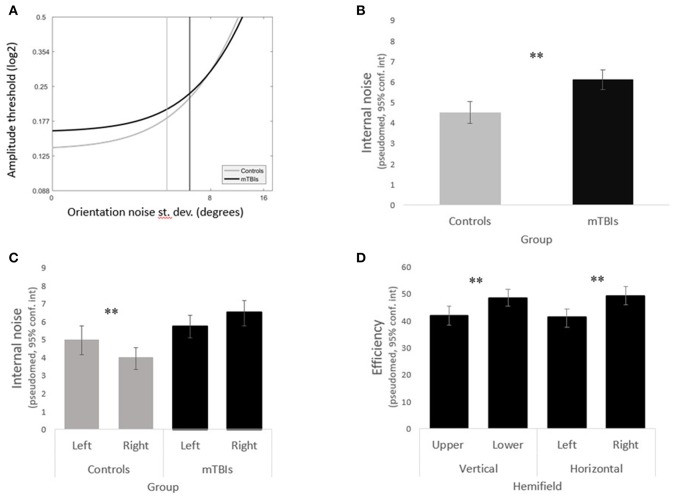
Main results. **(A)** LAM functions for the mTBI subjects (in black) and the control subjects (in gray). **(B)** The mTBI group shows significantly higher internal noise than the control group. **(C)** The left and right hemifields varried significantly in internal noise in the control group but not in the mTBI group. **(D)** mTBI subjects had significant biases in efficiency across both the vertical and horiontal hemifields.

Internal noise was significantly higher in the mTBI group than in the control group (Figures 3A,B) (*WTS* = 8.64, *p* = 0.003). We noted a significant interaction in the visual field biases between group and horizontal hemifields (*WTS* = 7.97, *p* = 0.005). Control subjects had lower internal noise than mTBI subjects (in both horizontal hemifields) with even lower internal noise in the right hemifield than in the left.

Analyzing the data within groups, we found that control subjects presented a significant horizontal bias (*WTS* = 4.86, *p* = 0.03) with lower internal noise in the right hemifield as opposed to mTBIs who did not have any hemifield bias in internal noise (horizontal *WTS* = 2.78, *p* = 0.09, vertical *WTS* = 3.43, *p* = 0.06) (Figure 3C).

### Abnormal Efficiency Distribution Across the Visual Field Following TBI

Although there was no group difference in efficiency overall (*WTS* = 0.85, *p* = 0.36), efficiency remained constant across hemifields in the control group (all *p*'s > 0.1) whereas mTBI subjects presented significant horizontal and vertical biases (horizontal *WTS* = 12.52, *p* = 0.0004, vertical *WTS* = 11.78, *p* = 0.0006) with higher efficiency in the lower right quadrant (Figure 3D).

### Consistent Visual Field Quadrant Ranking After TBI

To assess potential visual field imbalances caused by mTBI, for each subject we rank-ordered the quadrants in terms of internal noise and efficiency (separately) and analyzed these rank scores using the same non-parametric method described above. The mTBI group exhibited visual field biases as measured by rank of both efficiency and internal noise (efficiency *WTS* = 20, *p* = 0.0002, internal noise *WTS* = 9.5, *p* = 0.23); this was not observed in the control group (efficiency *WTS* = 0.3, *p* = 0.96, internal noise *WTS* = 2.15, *p* = 0.54).

mTBI subjects presented significant horizontal and vertical biases (*WTS* = 9.9 *p* = 0.0016; *WTS* = 9.1 *p* = 0.002, respectively) in efficiency, and a significant vertical bias (*WTS* = 5.74 *p* = 0.017) in internal noise. Control participants presented none of these biases.

### Efficiency and Internal Noise Positively Correlated in Both Groups

To understand the dynamic relationship between efficiency and internal noise, we tested for correlations between these parameters for each individual quadrant in each group and found a significantly positive correlation between internal noise and efficiency in all quadrants for both groups (Spearman, *rho* > 0.4, *p* < 0.001). However, when looking at the parameters averaged across quadrants this positive correlation was only maintained in the mTBI group (Spearman, *rho* = 0.36, *p* = 0.01). All participants tended to have higher efficiency in the quadrants where they also exhibited higher internal noise, but only participants from the mTBI group compensated for higher internal noise with higher efficiency when all quadrants were taken into account.

### Internal Noise, Efficiency, and Visual Dysfunctions Reports

To investigate the relationship between the internal noise and efficiency parameters as measured by our task and the symptoms experienced by the patients, we tested whether their answers to the Visual symptoms questionnaires were correlated to their internal noise and efficiency. Interestingly, changes in visual habits were inversely correlated to the efficiency on the good-continuity discrimination task (Spearman, *rho* = −041, *p* = 0.04), meaning that the more patients made changes to their visual habits (screen time, reading, driving…) the less efficiency they exhibited at discriminating between valid and invalid contours. Although the strength of the correlation was small, this would suggest that patients who adapted their behavior to their visual impairments also showed less efficiency in using the available orientation information to render a perceptual decision.

### Time Since Injury

Studies looking at post-concussive symptoms typically span their data collection between the time of injury and the following year, finding a decrease when comparing time points (41–43). Surprisingly, when we tested whether internal noise or efficiency on the good-continuity discrimination task were correlated with the time elapsed since injury, we did not find any significant relationship (Spearman, *rho* < −0.1, *p* > 0.4). We did not find any relationship between the time since injury and any of the neuropsychology tests either.

### Multiple Concussion Participants

For the five multiple concussion participants that were tested on the contour discrimination task, internal noise was marginally higher than in the controls (*WTS* = 3.76 *p* = 0.053), and not different from the single mTBI group (*WTS* = 0.27 *p* > 0.6). When analyzing all three groups at once (Table 2), we found a significant main effect of Group on internal noise (*WTS* = 9.9 *p* < 0.007), as well as a significant interaction between the factors Group and Quadrants (*WTS* = 37.8 *p* < 0.000001). The multiple TBI group had higher internal noise (pseudo-median = 6.69°, conf.int = 5.29°-8.56°) than the single TBI group (pseudo-median = 6.1°, conf.int = 5.65°-6.58°), and the control group had even lower internal noise (pseudo-median = 4.51°, conf.int = 3.99°-5.06°).

**Table 2:** Result summary.

**Single mTBI**	**Polytrauma**
Higher internal noise than controls	Higher internal noise than controls and single mTBIs
Abnormal efficiency distribution across visual field	No effect found on efficiency

Efficiency of the multiple TBI patients did not vary compared to either group separately (controls/multiple TBIs *WTS* = 1.12 *p* > 0.2, TBIs/multiple TBIs *WTS* = 0.45 *p* > 0.4). When the data from the three groups were combined into a single analysis, neither Group nor Quadrants had a significant relative effect on efficiency (*WTS* = 1.5 *p* = 0.48; *WTS* = 2.2 *p* = 0.53, respectively), but there was an effect of the interaction of Group and Quadrants on efficiency (*WTS* = 20.8 *p* = 0.002).

## Discussion

Cortical integration is understood to be at the heart of many cognitive symptoms related to attention and memory following mTBI—a large network of cortical regions is engaged to carry out these fundamental cognitive processes (15, 16, 44). Directly measuring impairments of cortical integration is a serious challenge, because of the absence of informed quantitative models that fully capture the two crucial limiting factors, namely cortical integration and internal noise. By utilizing contour integration—a fundamental step in visual shape recognition that is well-characterized in terms of cortical integration (20, 45)—within the framework of the equivalent noise technique, we were able to overcome the limitations posed by cognitive measures while assessing changes in visual processing following mTBI.

We have discovered that mTBI may not actually result in less efficient cortical integration *per se*, but rather in increased internal noise. This is a first quantitative characterization of the post-TBI changes using a model-driven behavioral task (46–50). Our results also corroborated the previous finding of visual field biases being affected by mTBI—we found that cortical integration efficiency was different between the vertical and horizontal hemifields. Finally, we found that poorer cortical integration efficiency was correlated with greater change in visual habits of mTBI patients.

We observed a significant increase in internal noise despite the recognized variability in the mTBI population (51, 52), suggesting that internal noise is a valuable and valid construct in describing the visual processing changes that occur in this disorder. Occipital injury was not a common mode of insult, yet the bulk of the group exhibited elevated internal noise on a visual task. Adding to the emerging scientific evidence for cortical visual impairments following mTBI (11–14, 22), our experiment relied on a non-invasive psychophysical method to probe cortical errors and inefficiencies in the low- to mid-level visual areas of the human brain (20, 45, 53).

### Cortical Integration During Contour Perception

Contour integration is a basic building block of visual perception, and yet, it requires complex and balanced interactions (18, 19, 54, 55). This integrative process can be effectively probed using simplified stimuli consisting of colinear Gabor elements along a path defining a shape—in our case, a simple arc. Such colinear sets tend to pop-out against a background of randomly-oriented Gabors, as captured by the Gestalt rule of Good Continuation (29, 56, 57). Thus Good Continuation is the fundamental feature of a contour perception, and the task used here (21) directly measures this key aspect of visual perception.

The perception of a contour is not instantaneous (19, 58, 59) suggesting multiple levels of computation, and recent evidence suggests at least two major steps are involved—a first step where the individual elements are detected by V1 neurons and a second step where secondary connections (lateral in V1 and/or feedback from extrastriate areas) “fill-in” the gaps between the Gabor elements (20). In other words, the individual Gabor elements of a synthetic contour each have distinct cortical representations in the retinotopic map of V1 (55). These individual cortical representations then interact and integrate into a new form—the full contour—thus describing a simple and elegant example of cortical integration that can be tightly controlled via stimulus manipulations.

Although there are diverging views regarding the cortical mechanisms involved in contour perception, namely if linking between stimulus elements is explicit or not (18, 60, 61), some form of integration remains unavoidable, whether it follows a step-by-step summation or an algorithmic overlap of orientation and template filters across hierarchical processing levels. We propose that contour integration can serve as an effective model of cortical integration, because the individual elements of a contour have distinct cortical representations and because the integration of the contour requires pooling and interactions across a set of such cortical nodes. The magnitude of these interactions can be controlled by stimulus parameters such as collinearity, gap, and path curvature (62–65), unlike cortical interactions engaged in complex cognitive tasks. Given the tight control that is granted by stimulus manipulations on this well-characterized integrative cortical process, contour integration is an effective and efficient method of probing complex interactions in the injured brain.

### Cortical Visual Deficits After TBI

We had previously speculated that long-range fibers—i.e., those that integrate information across visual fields and cross at the corpus callosum—are most vulnerable to injury in mTBI (12). We and others (12–14, 66) have documented several changes to cortically-mediated visual processes after mTBI. Traumatic brain injury results in decreased contrast sensitivity across spatial frequencies, especially for second-order modulated patterns (13). Binocular disparity perception is also affected by mTBI (14), in addition to inter-ocular signal propagation (12). That contour perception is also affected by mTBI suggests multiple components of the ventral visual pathway, needed for shape and object analysis, may be affected by mTBI. In contrast, motion perception—putatively subserved by the dorsal visual pathway—is not affected in mTBI patients (67). The emerging pattern from these results is that the ventral visual pathway may be more vulnerable to injury, and more studies are needed to assess this possibility.

### Visual Field Biases After TBI

We speculate that the vertical bias (greater efficiency in the lower visual field) may be related to the importance of this hemifield for shape perception—Schmidtmann et al. (68) have reported that while on orientation discrimination tasks performance is balanced between the upper and lower hemifields, there is a distinct advantage in normal individuals in discrimination of complex shapes in the lower visual field. We build on this finding to suggest that perhaps following mTBI, patients increase efficiency selectively in the lower visual field because of its importance to shape recognition, as a compensatory effort.

The left-right bias is admittedly more difficult to explain, but a clue may lie in the bias already present in the normal controls—internal noise is significantly lower in the right hemifield. We did find that this bias is eliminated by mTBI. We speculate that this bias may be part of normal visual processing, and its disruption by mTBI may be compensated by a biased increase in efficiency corresponding to our observations.

### Internal Noise and Neural Noise

The concept of noise utilized here—internal noise, captured as a Gaussian random variable within the Lam model—can be understood as a generalization of multiple sources of neural noise including spike-timing variability, synaptic noise, membrane potential variability, etc (69). Complex circuits of neurons would likely exhibit complex noise properties that are not linearly related to the noise within individual units (70–72).

The concept of internal noise, as measured by the equivalent noise technique (48, 50) has been effective at capturing a variety of phenomenon that were previously understood as limited by processing capacity or sensitivity, including contrast sensitivity (47), attention (46), and cortical blindness (73). Previous studies had described the observed changes as a modulation of performance capacity or sensitivity but estimates of internal noise within an equivalent noise framework revealed performance was noise-limited not capacity-limited, highlighting the value of a generalized measure of internal noise in characterization performance changes.

A key component of the LAM is the distinction between internal noise and efficiency—the latter denoting the capacity of the system to utilize all the available information. In the present contour task, efficiency has a simple interpretation: it is an estimate of the capacity of the integrative cortical process to pool orientation signals across the retinotopic map to give rise to a coherent representation of the contour. In this respect, mTBI patients did not differ from controls, suggesting that cortical integration is not affected by putative injury.

### Neurophysiological Basis of TBI

TBI results in an array of changes to the brain physiology, including axonal injury (74–78), neuronal death (79–81), neurotransmitter rebalance (82–84), glial activation (85), vascular changes (86, 87), and cortical spreading depression (88–90), amongst other factors. Any of these factors would be expected to affect performance on a complex task such as ours. Thus, it is exceedingly difficult to relate the neurophysiological changes that accompany TBI to any aspect of performance on our task.

Crucially, however, participants performance for *integrating* information during contour perception is not what was affected by mTBI—mTBI seemed to only inject noise in this integration mechanism. Compensatory mechanisms activated after TBI maintained a similar degree of cortical integration, thus keeping neural circuits and networks intact, but at the cost of added noise. This is in contradistinction to the notion that tissue loss after TBI causes capacity loss—we speculate that post-TBI compensation seeks to minimize loss of connectivity and circuitry, and the observed deficits are not due to loss of network interactions, but due to increased noise in those interactions.

We did not select participants following the location where the head was hit, nor did we aim to specifically recruit patients who suffered from torsion, direct hit, or indirect jolt, meaning that our cohort included a wide range of mild TBI type. Because none of our participants had any brain lesion (to the visual system or otherwise), heightened internal noise is a general consequence of mTBI stemming from a diffuse cortical imbalance that cannot possibly be restricted to the visual system. We speculate that other sensory modules would be similarly affected by mTBI, and that the LAM could be adapted to capture an general perception internal noise profile.

One cortical location previously thought to be instrumental in the modulation of visual processing noise, namely the Frontal Eye Field (91), and modulation of FEF activity with non-invasive methods such as transcranial magnetic stimulation or direct current stimulation (92), may serve to modulate internal noise and, coincidentally, modulate attentional control effects as well.

### Limitations

Abnormal integrative noise levels are a hallmark of other clinical populations as well. In the Autism Spectrum Disorder for example, noise has been measured via psychophysical methods (93) as in the present study, and operationalized as intra-individual variability in evoked EEG (94) and fMRI responses (95). Crucially, studies that tie physiological and cognitive measurements together allow for stronger claims and more encompassing interpretations, as in the case of schizophrenia (96). As such, functional imaging data should build on our findings to uncover the neural correlates of visual representation internal noise. We found no effect of gender on any of our measurements, but our sample did exhibit a gender bias, and it will be important to include gender as a factor in future mTBI studies because TBI may have gender-specific effects (97).

Increased integration noise was not previously considered as an encompassing feature of mTBI. We therefore stress the value of this encouraging first step toward understanding the functional mechanisms behind visual dysfunctions that follow mild Traumatic Brain Injury.

## Conclusion

In conclusion, we have demonstrated that cortical integration following mTBI is limited by abnormally high levels of internal noise as measured by our contour integration task, and that efficiency levels are not altered except in terms of visual field biases, possibly as a compensatory mechanism.

## Ethics Statement

All participants gave their informed written consent prior to taking part in the experiment. All procedures were in accordance with the Code of Ethics of the World Medical Association (Declaration of Helsinki) and were approved by the Research Ethics Board of the McGill University Health Center.

## Author Contributions

TR screened and tested participants, ran statistical analysis, and contributed to writing the manuscript. RF designed the project and contributed to editing the manuscript. AB designed the experiment and contributed to the statistical analysis and manuscript review. DS contributed to data collection and experiment design. RH co-designed the project and contributed to manuscript review.

### Conflict of Interest Statement

The authors declare that the research was conducted in the absence of any commercial or financial relationships that could be construed as a potential conflict of interest.
